# European agrifood and forestry education for a sustainable future - Gap analysis from an informatics approach

**DOI:** 10.12688/openreseurope.17205.3

**Published:** 2025-01-15

**Authors:** Stephen Burleigh, Håkan Jönsson

**Affiliations:** 1 Department of Process and Life Science Engineering, Division of Food and Pharma, LTH, Faculty of Engineering, Lund University, Lund, Skåne County, SE-221 00, Sweden

**Keywords:** agrifood, forestry, higher education, skills inventory, informatics, web-scraping

## Abstract

**Background:**

The NextFood Project ( www.nextfood-project.eu) started work in 2018 to identify ‘Categories of Skills’ that students should be equipped with to address the upcoming global challenges within agrifood and forestry disciplines. The skill groups are Innovative Mindset, Life-Long Learning, Network-Building, Strategic Management, Sustainability, Systems Perspective, Technological Knowledge and Versatility. This study is based on the initial skills and competencies mapping, but takes a different methodological approach. Instead of investigating what the research literature and professionals think are important skills for the future, this study asks how existing education programmes include or exclude these skills in the description of their programs.

**Methods:**

Python-based web-scraping scripts were used to collect texts from a selection of European Masters program websites, which were then analysed using statistical tools including Partial Least Squares regression and network analysis. A total of 14 countries, 27 universities, 1303 European Masters programs, 3305 web-pages and almost two million words were studied using this approach.

**Results:**

While agrifood and forestry Masters programs were aligned with the NextFood Project ‘Categories of Skills’ equal to or more often than unrelated Masters programs, we found evidence for the relative underuse of words associated with networking skills, such as collaboration, communication and eamwork. Agriculture-related programs used these words the least among the agrifood Masters programs. In contrast, agrifood programs used words associated with sustainability and system thinking more than the non-agrifood Masters programs.

**Conclusions:**

The informatics approach provides evidence that many European agrifood and forestry Masters programs are following the educational paths for meeting future challenges as outlined by the NextFood Project, with the possible exception of networking skills. This approach allows a complementary and time-efficient overview of the current state of education in the agrifood system in Europe.

## Plain language summary

European food production and agroforestry have many challenges to overcome in light of growing populations and global climate change. The NextFood Project ( www.nextfood-project.eu) started work in 2018 to identify ‘Categories of Skills’ that Masters-level students should be equipped with to address these upcoming challenges, including concepts such as sustainability, technological adaptation and networking. These skills were identified through many interviews and surveys of professionals within agriculture, food and forestry disciplines. To see if European agrifood and forestry Masters program websites also used vocabulary associated with these skills, which would be an indication that our educational system is preparing to meet these future challenges, we studied websites from a total of 14 countries, 27 universities, 1303 European Masters programs and almost two million words. We found that most programs used much of the vocabulary associated the recommended ‘Categories of Skills’, with the exception of the skill set ‘networking’ (with key concepts such as collaboration, sharing and teamwork). These results may suggest an underemphasis of networking skills in current Masters-level education within agrifood and forestry.

## Introduction

To secure a sustainable and resilient food system in a world facing unprecedented population growth and pressure on the ecosystems, it is crucial to meet the 2030 sustainable development goals developed by the United Nations. These diverse goals span human social and economic activities including how we produce food and sustainable agricultural and forestry practices. The training of future agrifood professionals is key to reaching these goals and therefore agrifood education programs must have the appropriate educational content necessary to cope with these coming challenges, including with new market conditions and novel technologies ( Charatsari *et al.*, 2020).

The role of education and potential shortcomings in the transformation towards sustainable and resilient agrifood systems has been discussed in several research disciplines in the last decades. As early as 1993, Newcomb (1993) proposed including leadership programs, extension education, agricultural communication and international development curricula to university agricultural education. Much of the current literature discusses the types of skills deemed necessary for facilitating a transition to sustainable the transformation and the difficulties arising in this process are explained on account of lacking the right type of skills. Charatsari and Lioutas (2019, 233) argue, for example, that, while “farmers exhibit high levels of empirically generated knowledge, they often lack the skills needed to adapt this knowledge into the new and complex circumstances which arise during and after conversion” to sustainable agriculture. They stress the importance of cultivating ‘soft skills’, including communication, collaboration, consultancy, and interpersonal skills ( *e.g.*, people skills, emotional intelligence, etc.). Other scholars have also identified network-building capacities ( Lamichhane *et al.*, 2016), facilitation expertise ( Moschitz *et al.*, 2015), entrepreneurial monitoring and co-innovation ( Dogliotti *et al.*, 2014), and guidance capabilities ( Wezel *et al.*, 2014), to name a few.

The complexities of a sustainability transformation have been widely acknowledged and several strategies have been suggested, including interdisciplinarity ( Bryant *et al.*, 2014), to develop systems thinking ( Hilimire *et al.*, 2014), experiential learning ( Ahmed *et al.*, 2017), promoting collective action and encourage critical reflection and self-reflection ( Valley *et al.*, 2018). A systemic and holistic approach has been argued to facilitate transformative learning in farming ( Francis *et al.*, 2017), rural development ( Wals *et al.*, 2004), and agronomic research and education ( Vietor, 2018). We can see how these new strategies have been implemented in education by the introduction of food studies ( Hamada *et al.*, 2015), agroecology ( Lieblein *et al.*, 2012), critical food systems education and education for food sovereignty ( Mann, 2019). Teaching and learning practices have moved beyond conventional pedagogy, towards more interactive and student-centred learning loops, including self-assessment ( Francis *et al.*, 2016; Galt *et al.*, 2013), using multiple sources of learning rather than traditional textbooks and introduce participatory learning by involving farmers and other actors from the agrifood supply chains in teaching ( Ahmed *et al.*, 2017; Šumane *et al.*, 2018). Such forms of participatory learning ( Pretty, 1995), where learning and innovation networks develop, is a possible way towards more sustainable agriculture and food systems.

### The research context

**Figure 1.**
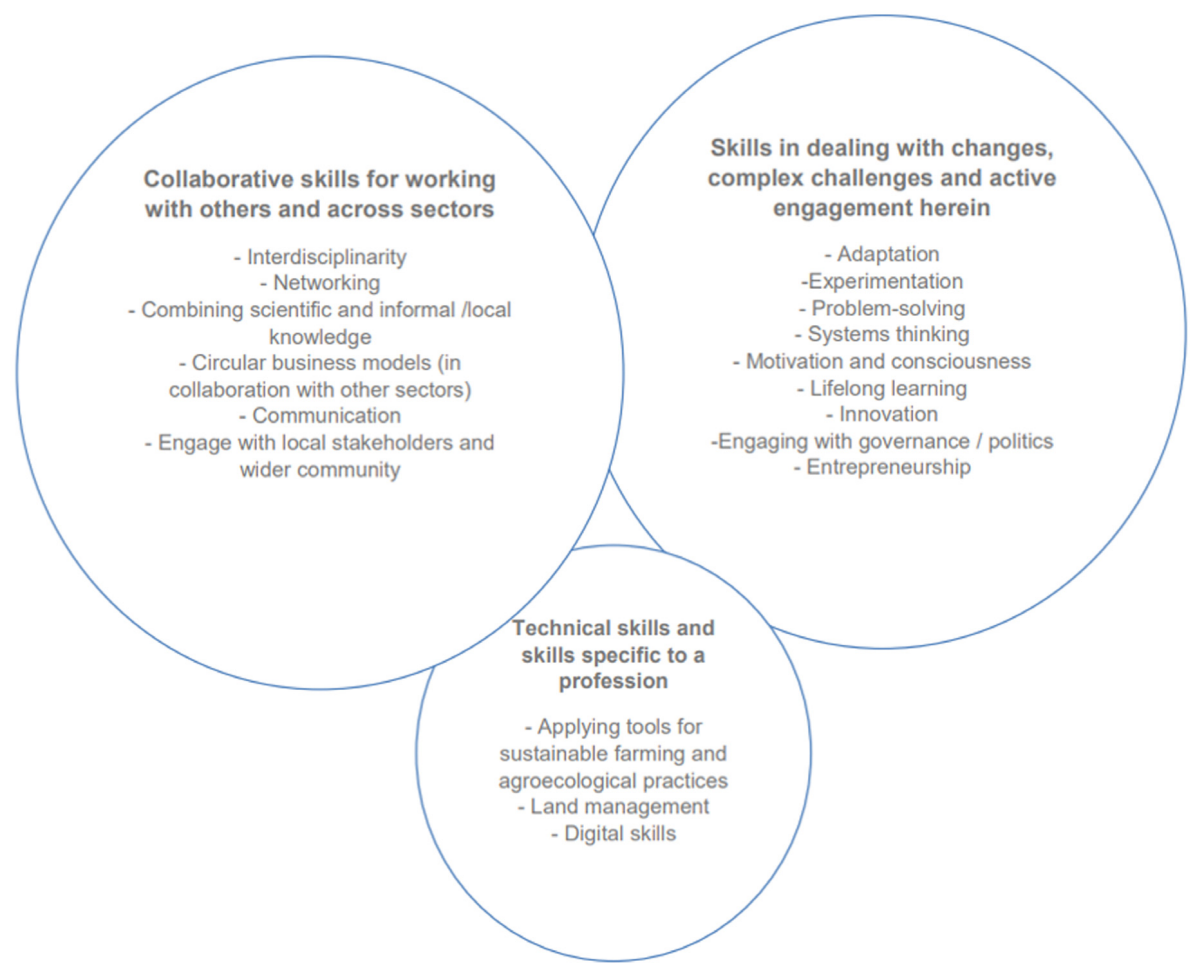
Future skills highlighted across the four datasets in the initial mapping of skills and competencies ( Rosenlund Hansen *et al.*, 2019: 36)

As a major funder for research and innovation, the European Commission launched a call in 2017 to build a research-based foundation for educating future professionals in the agrifood and forestry sector. The NextFood Project was funded and started work in 2018. As a first step an inventory of skills was made ( Rosenlund Hansen *et al.*, 2019). The inventory used four data sets; review of peer-reviewed literature, review of non peer-reviewed literature (reports, position papers) focus group interviews with professionals, practitioners and academics in the agrifood and forestry system, and a questionnaire survey. The results from the four data sets were compared to each other in order to identify the skills emphasized across the data. This resulted in three overarching sets of skills with subcategories ( Figure 1.)

Other deliverables from the NextFood project proposed additional skillsets, such as life-long learning tools ( Viaggi *et al.*, 2019; Viaggi *et al.*, 2021). The next step was to create an on-line audit tool that measures how an educational program or institution performs in relation to these skillsets to identify potential gaps in education ( Dimitrievski *et al.*, 2020).

This study is based on the initial skills and competencies mapping but take a different methodological approach. Instead of investigating what research literature and professionals think are important skills for the future, this study asks how existing education programmes include or do not include these skills.

The goal was to develop complementary methodologies to evaluate and follow up how appropriate higher education in agrifood and forestry was in coping with sustainability challenges. The aim of the study was to get a quantitative and non-targeted picture of to which extent European agrifood education was already addressing and educating towards what the NextFood mapping has concluded was important future skills. We designed a web-scraping based informatics study, which is a practice that has come of age, at least in computer science higher education ( Lunn *et al.*, 2020), to answer two questions: Is European higher education in the agrifood and forestry sectors using vocabulary associated with the NextFood ‘Categories of Skills’? Are there differences between educations directed to different sub-sectors, i.e., agriculture, food and forestry? Our strategy was to collect texts from a selection of European Masters program websites, identify the programs involved in the sectors of agriculture, food and forestry, determine how much the description of educations are aligned with these categories of skills and in what context, and how they compare to Masters programs not involved in agrifood sectors. From these analyses we draw some general conclusions about the current state of European agrifood education at the Masters level and identify possible gaps to be addressed in future curricula development.

## Methods

### Choosing Masters programs to study

The study was limited to Masters programs (ISCED 7 level) since they are a relevant educational level for most businesses in the agrifood sector and thus of strategic importance. Further, the Bologna Process, started in 1999, has streamlined higher education in Europe by setting common, comparable formats for the structure and learning outcomes of Masters programs. Candidate universities were selected from the European Tertiary Education Register database (ETER), filtered on the following criteria provided by ETER: Presence of Masters-level programs, > 10% STEM students and active graduate-level research carried out at the institution. These metrics are defined in the ETER database documentation.

Masters programs from the candidate universities were considered for study if they were described in English, had clearly defined program structures, were physically located at the corresponding university and had at least one page of text. Webpages considered for study included the main landing page, any linked pages to deeper program descriptions, up to the first two ‘student experience’ pages, course lists, pages describing specializations within the program and any program syllabi. Excluded pages included course syllabi, departmental pages, faculty pages, personnel pages and research pages not directly linked to the Masters program.

Once a list of candidate universities and their eligible Masters programs was compiled, they were grouped by country. Countries with few eligible Masters programs were excluded. The remaining countries were selected so that at least three countries were represented from Eastern, Northern, Southern and Western regions of Europe, respectively. While one goal was to include as many students in the study as possible by selecting universities with large student populations, several small universities nonetheless met selection requirements, including the University of Natural Resources and Life Sciences, Vienna Austria (BOKU) and the Swedish University of Agricultural Sciences, Alnarp Sweden (SLU).

### Web-scraping

**Figure 2.**
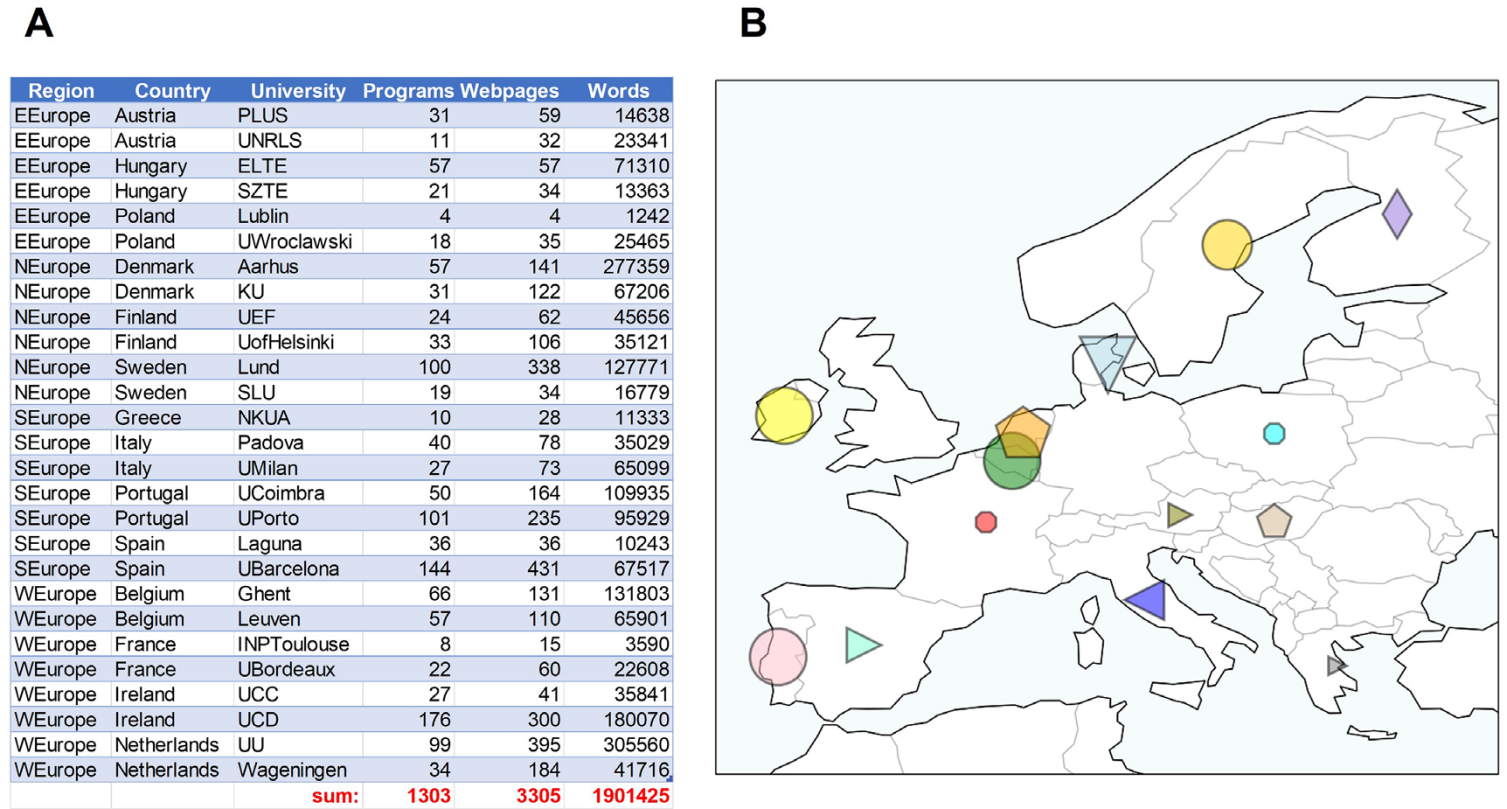
Regions, countries, universities, Masters program number, website number and words collected ( **A**). Map of the countries surveyed, each with its own symbol, with size representing the relative number of words collected ( **B**). Figure created using Python3.8 and the libraries ‘Matplotlib’ and ‘Cartopy’. Data available at the Zenodo repository ( Burleigh & Jönsson, 2024).

The Masters programs included in the study were web-scraped, cleaned of HTML, headers, footers, sidebars and non-program related information using the programming language Python3.8 and the library ‘BeautifulSoup’ (v.4). For the analyses, the texts were filtered to remove so-called ‘stop-words’ (common words such as ‘the’, ‘but’ and ’or’) using the Python library ‘NLTK’ ( Bird *et al.*, 2009), while keeping sentence structure. A total of 14 countries, 27 universities, 1303 Masters programs, 3305 web-pages and about 1.9 million stop-word-filtered words were collected ( Figure 2). Figures were made using Python3.8 and the library ‘Matplotlib’ ( Hunter, 2007), or GraphPad Prism (v.9). Website data was collected between January–March 2021.

### Identifying Masters programs associated with agriculture, food and forestry

In a few cases, the Masters programs were easy to identify as being associated with the sectors of agriculture, food and/or forestry based on their title, for example ‘EuroForestry’ from UNRLS Austria. However, most programs had less discernable titles, thus we identified the agrifood and forestry programs based on ‘domain-specific’ keywords found in their website texts. Programs that mentioned ‘agriculture’ and/or ‘agricultural’ at least once in their texts were categorized as being associated, at least to some degree, with the subject of agriculture, food-related Masters programs were those using the keywords ‘food’ and/or ‘foods’, while forestry Masters programs were identified as those using the keywords ‘forest’, ‘forests’ and/or ‘forestry’. The frequency of use for these keywords was calculated for each website.

### Choosing keywords associated with the NextFood ‘Categories of Skills’

As with the three sectors of agriculture, food and forestry, each Masters program was also scored for its association to the NextFood ‘Categories of Skills’. The categories and their keywords were derived from a number of NextFood deliverables ( Dimitrievski *et al.*, 2020; Rosenlund Hansen *et al.*, 2019; Viaggi *et al.*, 2019; Viaggi *et al.*, 2021), although since there was limited uniformity and harmonization in defining these ‘Categories of Skills’ between the various projects, we edited the categories to eight groups, with each skillset containing about 10 unique keywords. These categories and their keywords are presented in Table 1. For example, we defined the category ‘Versatility’ by the keywords ‘adapt’, ‘adaptability’, ‘adaptable’, ‘adaption’, ‘adaptive’, ‘flexibility’, ‘flexible’, ‘multiuse’, ‘resilience’, ‘resilient’, ‘versatile’ and ‘versatility’.

**Table 1.** Keywords used to define the eight educational themes; Innovative Mindset (‘Innov’), Life-Long Learning (‘LifeLearn’), Network-Building (‘Network’), Strategic Management (‘StratMan’), Sustainability (‘Sustain’), Systems Perspective (‘SysThink’), Technological Knowledge (‘TechKnow’) and Versatility. The sets of vocabulary were collected based on the NextFood qualitative skills inventory analysis, with some modification make each set unique. Word lists presented in alphabetical order.

Innov	LifeLearn	Network	StratMan	Sustain	SysThink	TechKnow	Versatility
create	assess	collaboration	leadership	sustain	circular	computer	adapt
creative	assessment	collaborative	management	sustainability	circularity	computers	adaptability
creativity	develop	collective	managing	sustainable	feedback	computing	adaptible
entrepreneurship	developing	communicate	organisation	sustaining	model	data	adaption
experimentation	development	communication	organisational	sustains	modeling	digital	adaptive
foster	experience	information	organization		modelling	digitalization	flexability
innovate	experiences	internet	orginizational		system	software	flexible
innovation	integrate	network	planning		systematic	technical	multiuse
innovative	integration	networking	political		systematically	technique	resilience
innovator	interdisciplinary	networks	politics		systems	techniques	resilient
pioneer	interpret	relationships	strategic			technological	versatile
startup	interpretation	sharing				technologies	versatility
startups	knowledge	teamwork				technology	
	learn						
	learning						
	plan						
	planning						
	skills						
	train						
	training						
	transdisciplinary						

### Normalizing the data to adjust for website size

Since there were clear differences in website size between Masters programs, raw counts of sector- and theme-related words from these website texts would have been problematic, since larger websites would have dominated the results, having more opportunity to use the targeted vocabulary, even though smaller sites might use this vocabulary more, relative to their word-count. Therefore, the study was carried out using normalized data, whereby counts of the sector- and theme-related keywords found in each of the Masters program texts were divided by the total words collected at the site. The 1303 Masters program websites, along with their normalized scores for the three sectors and the eight educational themes, are provided at the Zenodo repository ( Burleigh & Jönsson, 2024).

### Relations plot

Masters programs were plotted in relative proximity to their association (keyword counts) with each of the three sectors (agriculture, food and forestry) using Python3.8 and the libraries ‘Matplotlib’ and ‘NetworkX’ spring layout. Symbol size was based on the total number of keywords identified within the texts of each program. Programs symbols were colored by region (EEurope, pink; NEurope, Blue; SEurope, green; WEurope, orange).

### Box plots

To compare the sector-related Masters programs (agriculture, food and forestry) to those not associated with these sectors, we grouped the agriculture, food and forestry programs into a single ‘agrifood and forestry’ group, here termed ‘AgFoFor’, while Masters programs not associated with these sectors were grouped into an ‘Other’ group. Hence, the ‘Other’ group acted as a control, being comprised of hundreds of diverse, non-agrifood and forestry Masters programs and represented the current level of use of the vocabularies in question outside of the sectors of interest. Since the data was non-parametric (non-normal distribution due to most programs containing few keywords, while a minority contained many) as tested by Kruskal-Wallis statistics (data not shown), we presented the results in the form of box plots comparing median values instead of bar charts using averages.

### Partial Least Squares analysis

To study the relationships between Masters programs using keywords associated with agriculture, food, forestry and the eight educational themes, a Partial Least Squares (PLS) analysis was carried out using Matlab (v.2019b), whereby the agriculture, food and forestry Masters programs were used as explanatory variables, while the eight educational themes were defined as the response variables. In the case where we use Matlab in our study, we provide a suitable Python replacement program in the online repository ( GML at Lund University, 2024). In the plot, positive relations are those variables that are located near each other, while negative relations are those opposite and on the diagonal to each other. Variables farther from the center of the graph have greater impact on the results.

Further analysis in the form of an Effects table using Partial Least Squares Regression with jackknife resampling was carried out to identify what relations can be considered significant in the PLS plot. For our study, alpha was set at 0.05 and the first three principal components were used. A positive relation (a ‘1’ on the Effects table) for a given sector (agriculture, food or forestry) and educational theme (‘Innov’, ‘LifeLearn’, ‘Network’, ‘StratMan’, ‘Sustain’, ‘SysThink’, ‘TechKnow’ or ‘Versatility’) represents a significantly higher usage of vocabulary associated with that theme, relative to the two other sectors. A negative relation (a ‘-1’ on the Effects table) for a given sector (agriculture, food or forestry) and a given educational theme represents a significantly reduced usage of vocabulary from the theme, relative to the two other sectors.

### Validating our choice of keywords used to identify the sectors of interest

The study relied on a few select ‘domain-specific’ keywords to label Masters programs as having some association with the sectors agriculture, food and/or forestry. For example, any program mentioning ‘forest’, ‘forests’ or ‘forestry’ was labelled as having some relation to the forestry sector. However, given that this strict labelling method might be too exclusive, we therefore validated our results by repeating the study using a larger set of objectively-derived keywords to see if similar results could be obtained. We did this by picking the top 10–11 most frequent words (stop-word filtered, with minor edits to ensure uniqueness among the lists) found in Wikipedia website descriptions of each of the three sectors, agriculture, food and forestry. Thus, the validation test, as compared to the main study, used about five times more keywords and was based objectively on communal descriptions of the three sectors.

## Results

### Data structure

**Figure 3.**
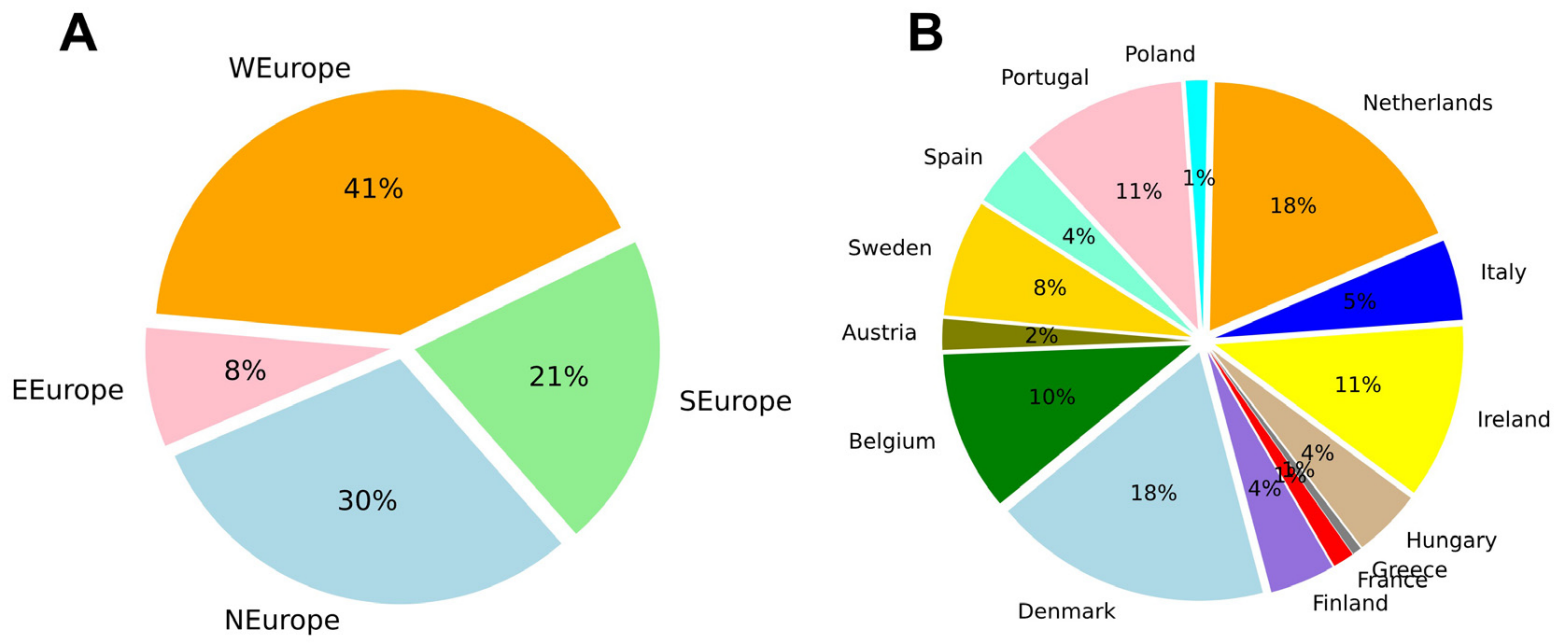
Relative number of words collected from Masters program websites distributed by region ( **A**) and country ( **B**). Based on 1,901,425 words collected from 1303 Masters programs. Plots made using Python3.8 and the library ‘Matplotlib’.

The words collected for each program were summed at the university, country and region level. Western Europe had the most words collected (41%), while Eastern Europe had the fewest (8%) ( Figure 3A). Denmark (18%) and the Netherlands (18%) had the most words by country, while Greece (1%) and France (1%) had the least ( Figure 3B). In the case of Greece and France, the limited number of words collected was due to relatively small websites and few programs described in English.

**Figure 4.**
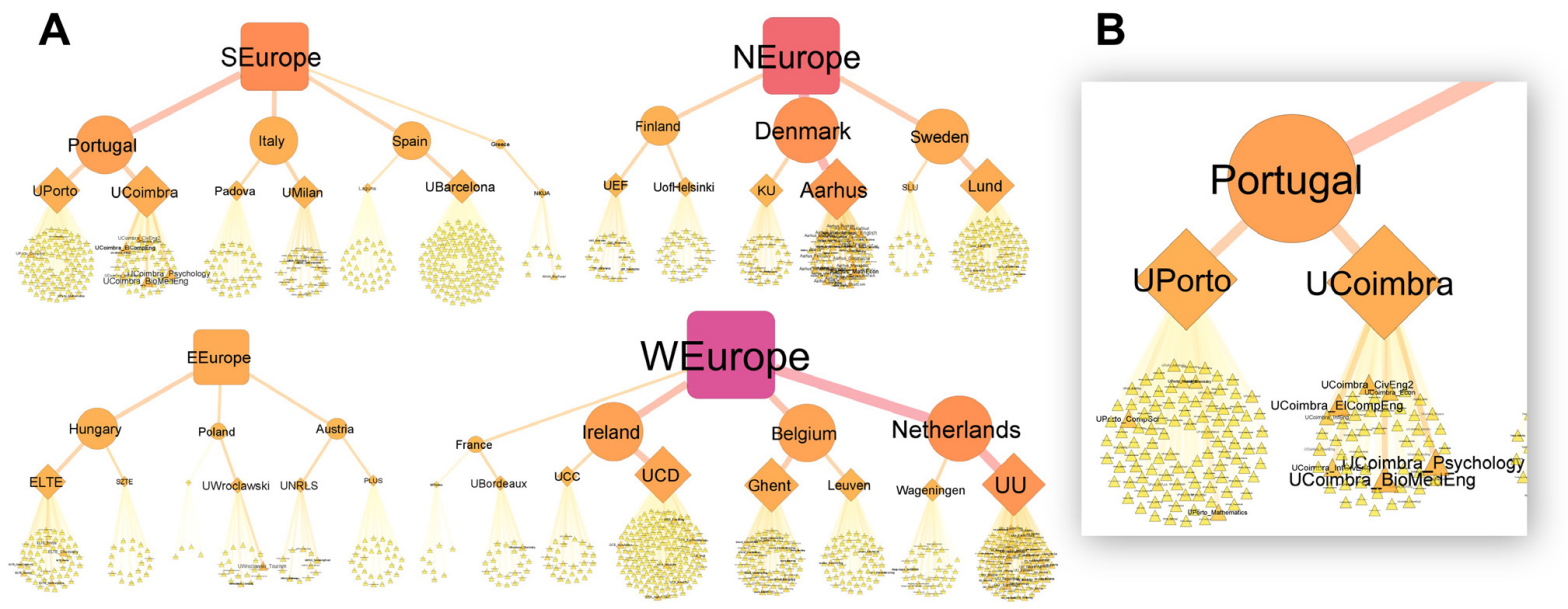
Source of the words collected at the Masters program, university, country and regional levels ( **A**). Masters programs, universities, countries and regions represented by triangles, diamonds, circles and squares respectively. Symbol size, label size and color (yellow to red transition) are relative to the total number of words collected. Based on 1,901,425 words collected from 1303 Masters programs. A closer view of Portugal’s network ( **B**). Made using Cytoscape (v3.9.0).

Due to the high variation in the number of words collected between Masters programs, universities, countries and regions, we used network analysis, a method for studying relationships and interactions within a system, to “show where the words came from” and view the overall structure of the data. Strong variation can be seen between universities in how many Masters programs were offered ( Figure 4A, the number of triangles per university) and how many words each program contributed (the relative size of the triangles). WEurope and NEurope had the largest vocabulary pools, while EEurope had the smallest ( Figure 4A, as seen by the relative size of the square symbols). Variation and complexity in the data can also be seen at the university level. For example, UPorto and UCoimbra both contribute a similar number of words to Portugal’s vocabulary pool (as evident by similar-sized university symbols), even though UPorto has considerably more Masters programs (more program symbols), while UCoimbra has fewer, yet more descriptive programs (fewer, but larger program symbols) ( Figure 4B).

### Masters programs involved in agriculture, food and forestry

**Figure 5.**
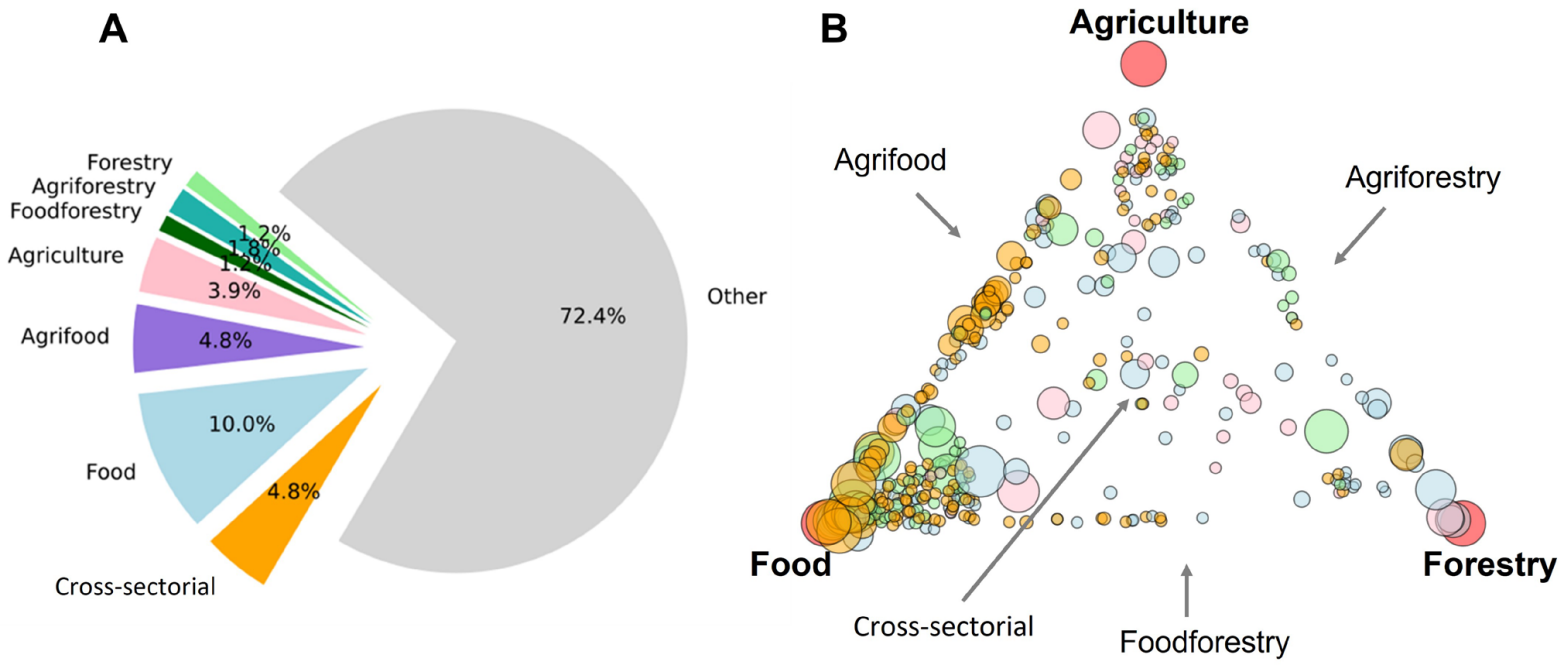
Agriculture, food and forestry Masters programs and their cross-sectorial combinations. The category ‘Cross-sectorial’ is defined here as those programs having some degree of association with all three sectors, while ‘Other’ represents Masters programs with no association to any of the three sectors. Piechart of program types ( **A**). Masters programs plotted in relative proximity to their association with each of the three sectors (in the corners, marked by red circles) ( **B**). Program symbol size is relative to the total number of keywords identified in their texts. Programs symbols were colored by region (EEurope, pink; NEurope, Blue; SEurope, green; WEurope, orange). Plots made using Python3.8 and the libraries ‘Matplotlib’ and ‘NetworkX’ spring layout with jitter (small, random variations) to separate overlapping datapoints.

Masters programs that used at least one word from one or more of the three sectors accounted for 360, or 27.6% of the 1303 Masters programs studied ( Figure 5A). Hence, about a third of the Masters programs studied had at least some association with agriculture, food and/or forestry. Of these 360 programs, 199 had at least one word from the agriculture keyword list, 269 for food and 117 for forestry.

Figure 5A breaks down the sectors of agriculture, food and forestry into subdivisions such as ‘agriforestry’ and ‘food-forestry’, based on shared keywords (for example a website containing both ‘food’ and ‘agriculture’ sector keywords). These Masters programs are relatively evenly distributed among the subdivisions, with the majority defined as ‘food-only’ programs (10.0%). Programs involved in all three sectors, here labelled as ‘cross-sectorial’, account for 4.8% of all Masters programs. Most Masters programs (72.4%) studied, however, had no association with any of the three sectors.

A more in-depth analysis was carried out using a relations plot ( Figure 5B). Here, each Masters program was positioned between the sectors of agriculture, food and forestry based on how many words they shared with each sector. Most programs were on or near the agriculture-food vertex, denoting programs involved to some degree in both agriculture and food, which is in strong contrast to forestry programs, which had fewer shared associations with the other sectors. It is apparent by color distributions that the region WEurope (orange circles) has abundant and well-described food and agrifood programs. The lack of balanced, cross-sectorial programs that have equal weights of all three sectors (programs located in the middle of the plot) is quite revealing and might suggest a relative shortfall of balanced agriculture-food-forestry programs in European higher education.

### Agriculture, food and forestry programs relative to other programs

**Figure 6.**
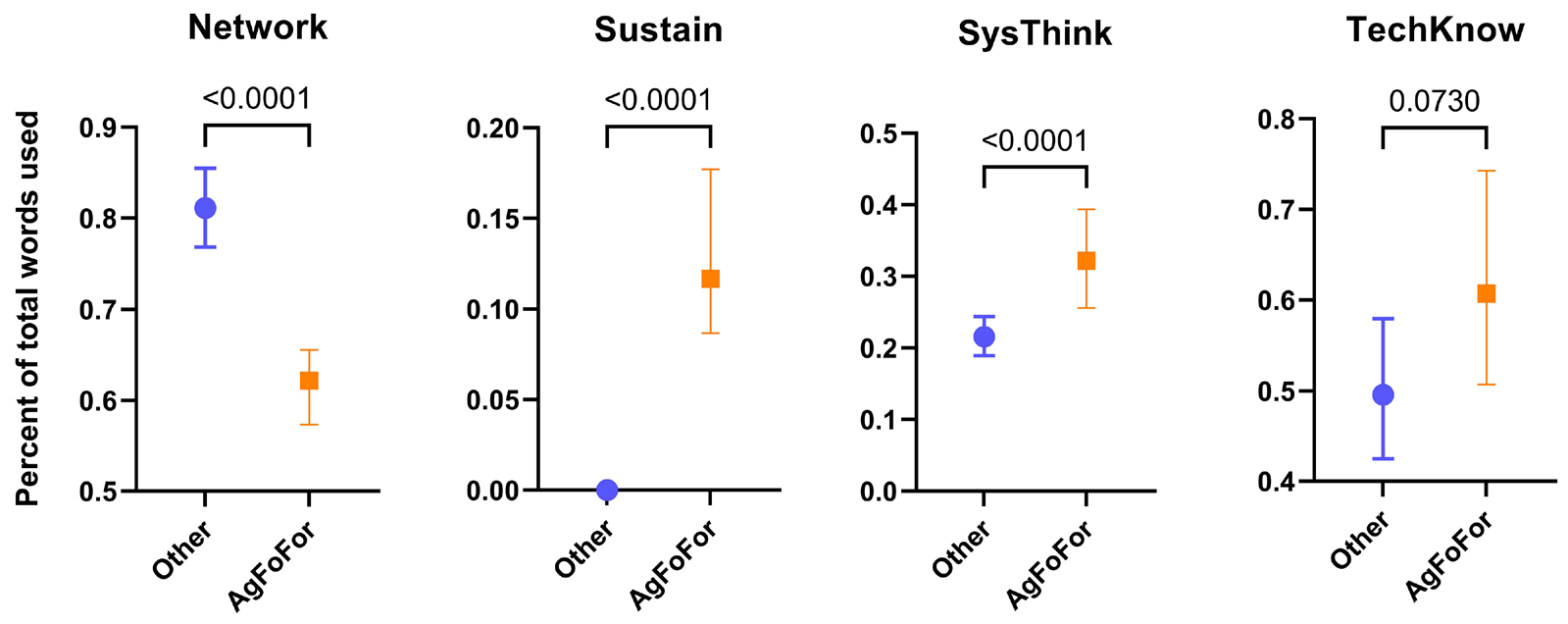
Use of the ‘Categories of Skills’ thematic vocabulary in European agrifood and forestry Masters programs. Median values of the percentage of vocabulary used from specific themes in agrifood/forestry Masters programs (AgFoFor) and unrelated Masters programs (Other). Based on 360 agrifood and forestry Masters programs involving 656504 words and 943 unrelated Masters programs involving 1,244,921 words. Error bars represent confidence intervals at 95%. P-values were determined by a non-parametric test (Kruskal-Wallis) using GraphPad Prism (v. 9).

To determine the extent to which agriculture, food and forestry Masters programs use vocabulary from each the eight educational themes, they were compared, as a group, to all other Masters programs. The agrifood and forestry group was termed ‘AgFoFor’, while non-agrifood and forestry Masters programs (representing 72.4% of the total Masters programs studied, see Figure 5A) were pooled into an ‘Other’ group. For three of the educational themes, Sustain, SysThink and TechKnow, the agrifood and forestry Masters programs used vocabulary more often than the non-agrifood and forestry programs ( Figure 6). However, for one theme, Network, with keywords such as ‘communication’, ‘collaboration’ and ‘networking’ (see Table 1), the agrifood and forestry programs used strikingly less vocabulary than the non-agrifood and forestry programs. The use of vocabulary from the themes Innov, LifeLearn, StratMan and Versatility were not significantly different between the two groups (data not shown).

### PLS plot and effects table

**Figure 7.**
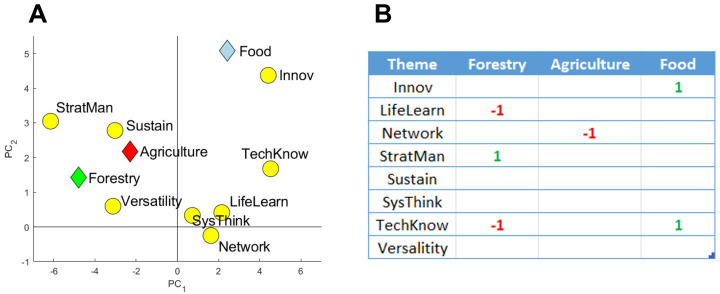
PLS plot ( **A**) and Effects table outcomes ( **B**) of relationships between agriculture, food and forestry Masters programs and the eight educational themes, the ‘Categories of Skills’. For the Effects table, alpha was set at 0.05 using the first three principal components. A ‘-1’ represents a significant negative relation, whereas a ‘1’ represents a significant positive relation. Results were cross-validated using jackknife resampling. Created using Matlab (v.2019b).

The PLS plot showed that agriculture, food and forestry Masters programs had strong associations with some of the educational themes ( Figure 7A). Food programs were positively associated with the theme Innov, while agriculture was associated with the theme Sustain. A clear, negative association can be seen between agriculture programs and the Network theme (located diagonally on the PLS plot). The theme SysThink appears to have no impact on the results, due to its proximity to the center of the graph, suggesting that the vocabulary from this theme is used more-or-less equally among the three sectors.

Further analysis in the form of an Effects table using Partial Least Squares Regression (cross-validated using jackknife resampling) was carried out to identify what relations can be considered significant ( Figure 7B). Several relations were identified and matched well with the associations seen in Figure 7A. The negative relation between agriculture and the Network theme implies that agriculture Masters programs use networking vocabulary significantly less than food and forestry Masters programs. Given that Figure 6 clearly showed that agrifood and forestry Masters programs, *as a group*, underused networking vocabulary when compared to other Masters programs, it can be concluded that all three sectors use less networking vocabulary relative to non-agrifood and forestry Masters programs, with the worst being agriculture.

### Contextual analysis of the educational themes

To better understand the context in which the educational theme vocabularies were being used in the agrifood and forestry website texts, we collected the words that both preceded and followed each keyword of each theme, creating phrases. These phrases were then summed and the most popular identified. Table 2 presents the top five phrases involving the eight educational themes in the texts of the agrifood and forestry Masters programs. Not surprisingly, many of the phrases included the words ‘agriculture’ and ‘food’, for example ‘sustainable agriculture’, ‘sustainable food’, ‘food systems’, ‘agricultural innovation’ and ‘food innovation’. However, none of the top phrases were found to be associated with any forestry-related words, which may reflect forestry’s rather limited educational footprint.

**Table 2.** Top five phrases involving the eight educational themes in the website texts of the agrifood and forestry Masters programs. ‘Word1’ and ‘Word2’ are the first and second word of the phrase, respectively, and ‘Cnts’ is the number of times the phrase was found in the texts. Based on 656504 words collected from Masters programs associated with agriculture, food and/or forestry.

Theme	Word1	Word2	Cnts	Theme	Word1	Word2	Cnts
Innovation	food	innovation	46	Sustain	sustainable	development	526
	agricultural	innovation	37		sustainable	agriculture	122
	innovation	management	32		sustainable	food	96
	innovative	solutions	27		sustainable	management	91
	applied	innovation	25		sustainable	energy	87
LifeLearn	sustainable	development	527	SysThink	food	systems	130
	internal	assessment	404		information	systems	110
	study	plan	276		circular	economy	75
	learning	outcomes	257		systems	analysis	74
	external	assessment	214		production	systems	71
Network	communication	skills	171	TechKnow	engineering	technology	311
	information	systems	110		data	analysis	232
	information	activities	92		food	technology	202
	information	technology	82		digital	technologies	102
	written	communication	67		data	collection	84
StratMan	resource	management	282	Versatility	adaptive	water	18
	project	management	244		flexible	profile	14
	water	management	196		ecosystem	resilience	6
	environmental	management	175		resilience	numerous	6
	spatial	planning	122		flexible	competitive	6

We also looked at the theme-associated phrases within each of the three sectors ( Table 3). The results mirror the PLS analysis, whereby agriculture programs, which had a strong association with the theme Sustain, used the phrase ‘sustainable development’ often in their website texts. Likewise, the food programs, which had a strong association with the theme Innov in the PLS analysis, used the phrase ‘food innovation’ often in their texts. Forestry Masters programs used phrases involving some of the TechKnow vocabulary such as ‘information technology’, which is surprising, considering the PLS Effects table suggested that forestry programs are rather weak in TechKnow vocabulary. Note that the theme ‘Life-Long Learning’ was left out of Table 3, since the keywords of LifeLearn, for example ‘skills’, ‘learn’ and ‘develop’ are so common in Masters program texts that phrases involving these ubiquitous words would dominate the top 10 list.

**Table 3.** Top ten phrases involving the keywords of the eight themes found in the text of agriculture, food and forestry Masters programs. ‘Word1’ and ‘Word2’ are the first and second word of the phrase, respectively, and ‘Cnts’ is the number of times the phrase was found in the sector-specific texts. Based on 656504 words collected from Masters programs associated with the sectors of agriculture, food or forestry.

Agriculture			Food			Forestry		
Word1	Word2	Cnts	Word1	Word2	Cnts	Word1	Word2	Cnts
sustainable	development	106	engineering	technology	308	cognative	modelling	20
information	systems	52	food	technology	107	forest	management	20
project	management	42	project	management	57	complex	systems	18
climate	systems	33	data	analysis	54	resource	management	16
geographical	information	31	spatial	planning	53	data	management	14
communication	skills	30	resource	management	51	support	systems	14
data	analysis	29	information	activities	48	information	systems	12
water	management	27	sustainable	energy	44	network	model	10
management	systems	26	food	innovation	43	informaiton	technology	8
systems	biology	26	information	technology	42	modelling	introduction	8

### Validating our choice of keywords to identify the agrifood and forestry programs

We tested whether using a minimalist set of ‘domain-specific’ vocabulary to label Masters programs as having a relation to our sectors of interest yielded similar results as when using a more complex list of sector-related keywords derived from a non-targeted approach ( Table 4). Interestingly, we observed few substantial differences between the original and validation study. The validation study had 1) more Masters programs associated with agriculture, food and forestry (32.6% *vs.* 27.6% for the validation and main study, respectively), 2) the agrifood and forestry Masters programs had a more significant relationship with technological knowledge relative to the ‘Other’ Masters programs (p=0.012 *vs.* p=0.073 for the validation and main study, respectively), 3) the sector of food was less associated with technological knowledge (no significant relation was seen in the validations study’s Effects table), 4) agriculture was more associated with sustainability (a significant relation was seen in the validation study’s Effects table) and 5) there were also some unremarkable changes to the top ten phrases involving the keywords of the eight themes ( Table 3).

**Table 4.** The validation study keywords to define the sectors of agriculture, food and forestry. The vocabulary lists were derived by web-scraping and selecting the most frequently used words from the Wikipedia websites on agriculture, food and forestry, respectively, with minor edits to avoid common and shared words. Websites were scraped using Python3.8 and the library ‘BeautifulSoup’ (v4), filtered of so-called ‘stop-words’ using the Python library ’NLTK’ and selected based on frequency of use. Word lists presented in alphabetical order.

Agriculture	Food	Forestry
agricultural	cook	afforestation
agriculture	cooking	deforestation
breed	diet	forest
breeding	diets	foresters
breeds	famine	forestry
farm	food	forests
farmers	foods	logging
farming	hunger	silviculture
farms	hungry	timber
livestock	nutrition	woods
	nutritional	

## Discussion

Our work should not be considered a comprehensive study of European Masters programs, since we only used programs described in English in order to keep the design as simple as possible. Indeed, our filters removed Germany from the list of countries studied, despite their central role in European education, due to the relative underuse of English in describing many of their Masters programs. A more comprehensive polyglot study is technically achievable, but due to the added complexity, should only come after this initial study using one language. Nonetheless, this study sampled both large and small European universities over broad geographic areas and different cultural regions.

We identified interesting and statistically significant relations between agriculture, food and forestry Masters programs and their alignment with the eight NextFood ‘Categories of Skills.’ However, our design does place limits on interpretation, since we only analysed the online *description* of the Masters programs and not the programs themselves. A more in-depth analysis could include the study of individual course descriptions and syllabi within select Masters programs to better understand and quantify these relations.

A general conclusion from the study is that, at least when it comes to the discursive presentations of Masters programs, the agrifood and forestry education in Europe has already moved in the direction of sustainability, along the paths identified in NextFood. If we compare this with the statements in the literature review that certain changes are needed, Europe may already be moving in the suggested direction. The systemic and holistic approach advocated by Francis *et al.* (2017) corresponds well with the strong use of systemic thinking in the vocabularies. Sustainability and technological skills are already in heavy use. The entrepreneurial and co-innovation skills that Dogliotti *et al.* (2014) suggested are at least not underperforming in relation to other educational programs, and the guidance capabilities ( Wezel *et al.*, 2014) and facilitation expertise ( Moschitz *et al.*, 2015) that can be covered under the term StratMan are also in line with other types of education. The main exception might be with the theme Network. Lamichhane *et al.* (2016) argued network-building capacities to be important, but we found that agrifood and forestry Masters programs underused the associated vocabulary relative to non-agrifood and forestry programs. The most common words from this educational theme were ‘communication’ and ‘information’, which on the other hand were among the “soft skills” that Charatsari and Lioutas (2019) recommended which may provide a good starting point for future discussions and curricula development to close this potential gap.

This study revealed or perhaps reiterated how dominant food-related Masters programs are relative to agriculture and forestry. Food programs were the most abundant type of agrifood program, had no obvious gaps in education as seen by the PLS plot and Effects table, had strong interdisciplinary relations with agriculture, and was strongly linked to the concept of innovation and technological knowledge. One concern, however, was the absence of the word ‘hunger’ in any of the main or validation contextual tests, which is surprising given that reducing hunger is one of the prominent food-related 2030 Goals. Hence, our results might imply that European Masters-level education in the food sector might focus on food issues for the ‘Global North’, while limiting other global food-related challenges ( FAO, 2017).

The two other sectors, agriculture and forestry, were less center-stage than food-related programs. Agriculture programs were often ‘agrifood’ programs, were associated with the theme Sustain, and commonly used phrases such as ‘agricultural innovation’ and ‘sustainable development.’ Agriculture may have a possible educational gap involving networking skills when compared to food and forestry programs. This potential gap is especially concerning, given that agrifood and forestry programs as a group underperformed relative to non-agrifood and forestry Masters programs in terms of using the Network-themed vocabulary. Forestry was strongly associated with the theme StratMan, but had limited cross-sectorial programs, few programs overall, and had a noticeable lack of keywords associated with the themes LifeLearn and TechKnow.

This study also showed that the theme Versatility as we defined it, is problematic. The associated vocabulary was not used often in the Masters program texts. While lack of use might be evidence for an educational gap across all the Masters programs studied, a closer look at the phrases involving the versatility theme showed that they were often contextless and uninformative, such as ‘flexible competitive’ and ‘resilience numerous’. The few exceptions were ‘ecosystem resilience’ and perhaps ‘adaptive water’. Such observations suggest that the theme Versatility might be a difficult ‘Category of Skills’ theme to define and quantify using our methods.

We validated our method of identifying agriculture, food and forestry Masters programs by comparing our main results, which were based on a minimalist set of selected ‘domain-specific’ vocabulary with the results from a second study that used a larger set of keywords not chosen by us, but instead indirectly derived by the academic community. We found that results were not substantially altered, suggesting that a few domain-specific key words were adequate for identifying our Masters programs of interest. This strategy avoids the creation of a long list of keywords that defines a given sector, but inadvertently adds subjective bias depending on who chooses the words. As for the educational themes, the ‘Categories of Skills’ keywords were subjectively selected by members of the NextFood Project and we make no claim that the ‘best’ keywords were chosen, nor even conclude that we studied all relevant categories. Indeed, it would be interesting to test other keywords, categories and concepts, such as keywords that might group ‘soft’ versus ‘hard’ agrifood and forestry skills.

There was much variation between Masters programs in terms of how much text could be collected by web-scraping, and this tended to correlate by country. As a result, we did not carry out any country-level comparisons and even our regional analyses were quite limited in interpretation. However, it would be informative to approach this study from a country and regional perspective, given Europe’s geographic, cultural and educational diversity, if the technical challenges of a multi-lingual study can be overcome.

## Conclusion

This article has demonstrated that a text-based, informatics approach can provide insight into the current state of agrifood and forestry higher education. It provides a foundation for fast and efficient diagnosis of how current education is aligned with policy goals. The results suggest that current education programmes are already using much of the vocabularies of the important skills for the future that was defined by literature and stakeholders in the agrifood sector. A notable possible exception was networking, a gap that can be addressed in future curricula development. This analysis can also be used as a starting point to study changes to European education over time. For example, conducting a new study in five or 10 years and comparing it to these results might provide fruitful trends. These follow-up studies can likely be assisted by AI tools that can process academic/scientific texts in different languages. Likewise, a breakdown of the control group, for example into social and natural sciences, could provide a deeper understanding of competences in other Masters-level programs. This study also emphasizes the importance for Masters program websites to use descriptive texts and relevant vocabulary when describing their educational resources and goals, as AI will likely use this data to assess some degree of program scope and quality. We addressed a number of limitations to our strategy including 1) unequal sampling of European regions, countries and their universities, 2) the analysis was based on the online *description* of the Masters programs, not the actual course content, 3) programs differed considerably in how much text they provided online and 4) the keywords chosen to define our sectors of interest may not have been optimal.

## Ethics and consent

Ethical approval and consent were not required.

The study follows the European GDPR guidelines. No personal information was disclosed in this study.

## Data Availability

Zenodo: Raw data for D1.1: Inventory of skills and competencies. https://doi.org/10.5281/zenodo.10782379 ( Burleigh & Jönsson, 2024). The project contains the following underlying data: -  extra_stopwords.csv -  keywords.csv -  website_data.csv Data are available under the terms of the Creative Commons Attribution 4.0 International license (CC-BY 4.0). Analysis code available from: https://github.com/GMLatLU Archived analysis code at time of publication: https://doi.org/10.5281/zenodo.10792373 ( GML at Lund University, 2024) License: The MIT License
