# Supplementary material for: European agrifood and forestry education for a sustainable future - Gap analysis from an informatics approach
Source: Open Res Eur. 2025 Jan 15;4:93. Originally published 2024 Apr 25. [Version 3] doi: 10.12688/openreseurope.17205.3 (PMC11757921; doi:10.12688/openreseurope.17205.3)
Supplement: Peer review history

# Reviewer report: Reviewer response for version 3

**DOI:** 10.21956/openreseurope.20945.r49806

**Published:** 2025-02-04

**Authors:** Mika Rekola

**Affiliations:** 1 University of Helsinki, Helsinki, Finland

The amendments from Version 2 are appropriate. They are mainly related to clarifying some concepts and removing typos.

 However, I do not see any response to my concern about the data. It would be necessary for anyone to be able to download the final dataset of 360 programmes. Now, the full data set of 1,303 programmes is provided. However, inside this data set it is not clearly indicated the sub-sample of 360 programmes.

 Please, respond to my report.

Is the study design appropriate and does the work have academic merit?

Yes

Is the work clearly and accurately presented and does it cite the current literature?

Yes

If applicable, is the statistical analysis and its interpretation appropriate?

Yes

Are all the source data underlying the results available to ensure full reproducibility?

Partly

Are the conclusions drawn adequately supported by the results?

Yes

Are sufficient details of methods and analysis provided to allow replication by others?

Partly

Reviewer Expertise:

higher education, pedagogy, forestry, forest education

I confirm that I have read this submission and believe that I have an appropriate level of expertise to confirm that it is of an acceptable scientific standard, however I have significant reservations, as outlined above.

# Reviewer report: Reviewer response for version 3

**DOI:** 10.21956/openreseurope.20945.r49803

**Published:** 2025-01-23

**Authors:** Roberto Henke

**Affiliations:** 1 Research Centre for Agricultural Policies and Bio-Economy, Council for Agricultural Research and Economics, Rome, Italy

For me the paper now is to be approved. I am pleased with their changes and their comments. Thank you for this opportunity.

 Best regards,

 Roberto

Is the study design appropriate and does the work have academic merit?

Yes

Is the work clearly and accurately presented and does it cite the current literature?

Partly

If applicable, is the statistical analysis and its interpretation appropriate?

Partly

Are all the source data underlying the results available to ensure full reproducibility?

Yes

Are the conclusions drawn adequately supported by the results?

Yes

Are sufficient details of methods and analysis provided to allow replication by others?

Yes

Reviewer Expertise:

Agrifood system economics and policies; Multifunctional agriculture and diversification in agriculture; Rural development, Agricultural and rural policy; Sustainability; Food policy

I confirm that I have read this submission and believe that I have an appropriate level of expertise to confirm that it is of an acceptable scientific standard.

# Reviewer report: Reviewer response for version 2

**DOI:** 10.21956/openreseurope.20243.r47534

**Published:** 2024-12-30

**Authors:** Md Manik Ali

**Affiliations:** 1 Hajee Mohammad Danesh Science and Technology University, Dinajpur, Rangpur Division, Bangladesh

This is an interesting study into the attention of agriculture, food and forestry master programs to future skill needs. The article presents an attempt to assess the alignment of the European Agri-Food and Forestry Master’s Programme with the competence categories identified by the NextFood project through a data-driven informatics approach.

 However, it suffers from methodological and analytical flaws that undermine its credibility and relevance. Relying solely on the descriptions of the programme websites is a weakness, as these descriptions do not accurately reflect the actual curriculum or educational outcomes. The selection of keywords in the article is a superficial approach to analyzing complex educational themes. By relying on a minimal set of keywords to represent multifaceted skills like sustainability, networking, or systems thinking, the study oversimplifies these concepts and risks misinterpreting their contextual relevance. For instance, words such as "collaboration" or "teamwork" are used as proxies for networking skills without accounting for how they are integrated into program descriptions or course content. Furthermore, the subjective selection of keywords, even when validated against Wikipedia-derived lists, introduces bias and lacks the rigor necessary for an objective analysis. This approach fails to account for the diversity of language, phrasing, and nuance used across different educational contexts, potentially leading to under- or over-representation of certain themes.

 A more robust methodology would involve a broader and more systematically derived keyword set, validated through expert consensus and tested across diverse educational texts, to ensure accurate and meaningful results. The exclusion of non-English programmes introduces significant regional and linguistic bias, which limits the representativeness of the results.

 Furthermore, the analysis of regional data is confounding due to uneven sampling and the fact that actual programme content, such as syllabus or outcomes, is not included in the analysis makes the conclusions speculative. Despite its limitations, the research lays a solid foundation for future studies, suggesting avenues for deeper analyses and long-term monitoring of educational progress in the agrifood and forestry sectors, while also emphasizing the importance of aligning education with global sustainability goals.

Is the study design appropriate and does the work have academic merit?

Partly

Is the work clearly and accurately presented and does it cite the current literature?

Yes

If applicable, is the statistical analysis and its interpretation appropriate?

Yes

Are all the source data underlying the results available to ensure full reproducibility?

Yes

Are the conclusions drawn adequately supported by the results?

Yes

Are sufficient details of methods and analysis provided to allow replication by others?

Yes

Reviewer Expertise:

Agroforestry

I confirm that I have read this submission and believe that I have an appropriate level of expertise to confirm that it is of an acceptable scientific standard, however I have significant reservations, as outlined above.

# Reviewer report: Reviewer response for version 2

**DOI:** 10.21956/openreseurope.20243.r46057

**Published:** 2024-12-17

**Authors:** Sara Burbi

**Affiliations:** 1 Institute of Plant Sciences, Sant’Anna School of Advanced Studies, Piazza Martiri della Libertà 33, Pisa, Italy

The work described in this article is based on the activities of the NextFood Project (2018-2022). It touches a very interesting topic, that is whether or not European agrifood and forestry Masters programmes are addressing the categories of skills identified by the project as necessary to face global challenges in the agrifood and forestry sector; and tests a text-based, informatics approach to obtain results. The rationale behind such a study is solid. The methodological approach is sound and provides interesting results from 27 universities in 14 EU countries. Concepts and skills associated with or contributing to networking and collaboration are the least found in Masters programme. More technical and applied topics related to agrifood and forestry sectors are addressed by a large number of programmes, suggesting the programmes are potentially in line with the technical requirements of courses targeting these sectors.

 However, despite these interesting results, it is recommended that some elements of the study are improved.

 Recommended revisions:

 1. With regards to data in English, it is not clear why the authors used the term 'agriforestry' when the widely used term is 'agroforestry'. A revision is highly recommended to ensure the correct terminology has been used in the study.

 2. The dataset available here: https://zenodo.org/records/10782379 is confusing. Some keywords mentioned in the article do not appear in the dataset, e.g., agriforestry / agroforestry. The website_data file contains a long list of unrelated programmes. It is recommended a note is added to explain the content of the data files.

 3. The authors rightly state the limitations of the study, in primis the use of only programmes described in English. However, the article also makes several generalisations about education in Europe inferred from the results of the study, despite having collected data from 14 countries only. This is rather contradictory. The discussion should be rephrased to avoid generalisations about the sector. The authors should ensure the focus remains on the testing of the text-based, informatics approach for such studies, which has great potential.

 Additional comments:

 - Article keywords should include 'agriculture'.

 - Chinese characters are replacing quotation marks. This should be rectified.

Is the study design appropriate and does the work have academic merit?

Yes

Is the work clearly and accurately presented and does it cite the current literature?

Yes

If applicable, is the statistical analysis and its interpretation appropriate?

Yes

Are all the source data underlying the results available to ensure full reproducibility?

Partly

Are the conclusions drawn adequately supported by the results?

Partly

Are sufficient details of methods and analysis provided to allow replication by others?

Partly

Reviewer Expertise:

Sustainable agriculture; Rural development; Agroecological transitions; Agroecology; Agroforestry; Participatory research; Transdisciplinarity; Higher education

I confirm that I have read this submission and believe that I have an appropriate level of expertise to confirm that it is of an acceptable scientific standard, however I have significant reservations, as outlined above.

# Reviewer report: Reviewer response for version 2

**DOI:** 10.21956/openreseurope.20243.r46781

**Published:** 2024-12-16

**Authors:** Mika Rekola

**Affiliations:** 1 University of Helsinki, Helsinki, Finland

Burleigh Stephen, & Jönsson Håkan. (2024). Raw data for D1.1: Inventory of skills and competencies [Data set]. Zenodo. https://doi.org/10.5281/zenodo.10782379

 Review report Dec. 16, 2024

 by Mika Rekola, University of Helsinki, mika.rekola@helsinki.fi

**ABSTRACT**

 Background

 The NextFood Project (www.nextfood-project.eu) started work in 2018 to identify ‘Categories of Skills’ that students should be equipped with to address the upcoming global challenges within agrifood and forestry disciplines, and involved concepts such as sustainability, technological adaptation and networking. This study is based on the initial skills and competencies mapping, but takes a different methodological approach. Instead of investigating what the research literature and professionals think are important skills for the future, this study asks how existing education programs include or exclude these skills in the description of their programs.

 Methods

 Python-based web-scraping scripts were used to collect texts from a selection of European Masters program websites, which were then analysed using statistical tools. A total of 14 countries, 27 universities, 1303 European Masters programs, 3305 web-pages and almost two million words were studied using this approach.

 Results

 While agrifood and forestry Masters programs were aligned with the NextFood Project ‘Categories of Skills’ equal to or more often than unrelated Masters programs, we found evidence for the relative underuse of words associated with networking skills, such as 舖collaboration舗, 舖communication舗 and 舖teamwork舗. Agriculture-related programs used these words the least among the agrifood Masters programs. In contrast, agrifood programs used words associated with sustainability and system thinking more than the non-agrifood Masters programs.

 Conclusions

 The informatics approach provides evidence that many European agrifood and forestry Masters programs are following the educational paths for meeting future challenges as outlined by the NextFood Project, with the possible exception of networking skills. This approach allows a complementary and time-efficient overview of the current state of education in the agrifood system in Europe.

 Keywords

 agrifood, forestry, higher education, skills inventory, informatics, web-scraping

**Reviewer COMMENTS**

 The article is about certain competencies that agrifood and forestry HE students should have after graduation. The study, based on website descriptions of programmes, is highly relevant and up-to-date. Data and methods are innovative and mainly very clearly and illustratively described. Results showing differences between program types and relations between programs are excellent and useful. My main concern is about selection of data and its description in the report and external data source.

 Abstract has some typos with wrong characters (both in online and pdf version): “networking skills, such as -

 舖collaboration舗, 舖communication舗 and 舖teamwork舗. Agriculturerelated”

 Please correct these to “ or leave with any character, the meaning is also clear without quotation mark “ symbol.

 Keywords should include “agriculture” and “food”.

 Method section describes how programs were selected: “Candidate universities were selected from the

 European Tertiary Education Register database (ETER), filtered on the following criteria provided by ETER: Presence of Masters-level programs, > 10% STEM students and active graduate-level research carried out at the institution. These metrics are defined in the ETER database documentation.” Please, provide here some more details. What is STEM criterion all about? What does this mean “Presence of Masters-level programs?

 Data is provided in the system called Zenodo.org, https://zenodo.org/records/10782379.

 A file “website_data.csv” has full data on 1575 lines of which 1303 are describing separate programmes (according to manuscript). Data variables are the following ones:

 ID                      Region              Country            University        Program                          Deptype           URL                   Text

 The critical issue is now the following question: Which of these 1575 data lines are concerning those 360 programmes that are representing agriculture, food, and forestry? There is a code “Deptype” which indicates programme type. This code includes, for instance, categories of agriculture and forestry. However, the classification principle describes on page 8, second column was “programs involved in agriculture, food and forestry Masters programs that used at least one word from one or more of the three sectors accounted for 360,”

 Having a look on data I found that some program that have keywords in their web-text were not classified as any of three relevant categories (agriculture, food, forestry) using this “Deptype” variable. For instance, a program under “Ecology” had a keyword “forest” in its webpage text and must be thus one of those 360 relevant programs. However, to my understanding there is no code indicating that in website_data.csv. As mentioned in the manuscript (p. 8, second column) these programs covered 27,6% of all programs. Therefore, the datafile has mainly non-relevant information that makes it impossible to replicate the study.

 Please, separate relevant data describing 360 programs out of full data (file website_data.csv) and  provide it online. That separated data should also has coding related to final relevant programs type: agriculture, food, forestry.

Is the study design appropriate and does the work have academic merit?

Yes

Is the work clearly and accurately presented and does it cite the current literature?

Yes

If applicable, is the statistical analysis and its interpretation appropriate?

Yes

Are all the source data underlying the results available to ensure full reproducibility?

Partly

Are the conclusions drawn adequately supported by the results?

Yes

Are sufficient details of methods and analysis provided to allow replication by others?

Partly

Reviewer Expertise:

higher education, pedagogy, forestry, forest education

I confirm that I have read this submission and believe that I have an appropriate level of expertise to confirm that it is of an acceptable scientific standard, however I have significant reservations, as outlined above.

# Reviewer report: Reviewer response for version 2

**DOI:** 10.21956/openreseurope.20243.r47948

**Published:** 2024-12-16

**Authors:** Roberto Henke, Giampiero Mazzocchi

**Affiliations:** 1 Centre for Policies and Bioeconomy, CREA, Rome, Italy; 2 Research Centre for Agricultural Policies and Bio-Economy, Council for Agricultural Research and Economics, Rome, Italy

The article deals with a very interesting topic, which is not often covered by scientific literature. High level education in the fields of agrifood systems is changing rapidly, opening the way to multidisciplinary (and transdisciplinary) approaches, sustainability issues, resilience, and new systematic approaches. The article investigates Masters programmes, comparing those specific in the fields of agri-food and forrest fields with neighbor disciplines and similar non-specific ones.

 I have only a few comments reported below:

 1) The introduction, in my point of view, does not present properly the topic and the bectives of the paper and remains quite generic, also ingenerating some confusion in the reader between the general issue of farmers (and other operators) education and training in agriculture with the specific issue of post-graduate agri-food secialistic masters programmes, which are not necesseraly and not often addressed to farmers.

 2) In figure 1 in the first circle I would refer also to transdisciplinarity as a new frontier of high level education, meaning crossing disciplinary bounderies, building a common language and vocabulary, setting common knowledge goals and integrating results in favour of the common more general  interests of science and society (see Morton et al. 2015 (Ref-1): Architectures of adaptive integration in large collaborative projects, Ecology and Society, 20(4)).

 3) Figure 2B is not very clear and there is no legends about the symbols used.

 4) In my opinion the article is a bit too self referenced to the results of the mother project (NextFood), while there is not much discussion about the results and the evidence compared to other literature. I imagine there is very scarce literature on the topic, but a little effort in framing the results in the debate and literature would improve the overall value of the paper. The same issue stands, in my opinion, with regards to the relevance and the generalisation of the conclusions of the paper.

Is the study design appropriate and does the work have academic merit?

Yes

Is the work clearly and accurately presented and does it cite the current literature?

Partly

If applicable, is the statistical analysis and its interpretation appropriate?

Partly

Are all the source data underlying the results available to ensure full reproducibility?

Yes

Are the conclusions drawn adequately supported by the results?

Yes

Are sufficient details of methods and analysis provided to allow replication by others?

Yes

Reviewer Expertise:

Agrifood system economics and policies; Multifunctional agriculture and diversification in agriculture; Rural development, Agricultural and rural policy; Sustainability; Food policy

We confirm that we have read this submission and believe that we have an appropriate level of expertise to confirm that it is of an acceptable scientific standard, however we have significant reservations, as outlined above.

## References

[1] : Architectures of adaptive integration in large collaborative projects. *Ecology and Society* .2015;20(4) : 10.5751/ES-07788-200405 10.5751/ES-07788-200405

# Reviewer report: Reviewer response for version 2

**DOI:** 10.21956/openreseurope.20243.r45635

**Published:** 2024-12-09

**Authors:** Jacques H. Trienekens

**Affiliations:** 1 Wageningen University, Wageningen, The Netherlands

This is an interesting study into the attention of agriculture, food and forestry master programs to future skill needs. My main comments are on the (structure and argumentation in) abstract and introduction, and on the methods section.

 Results, discussion and conclusion sections give interesting and new insights! Conclusions, limitations and further research are well explained and described.   

**Abstract**

 In the background paragraph three concepts are mentioned: sustainability, technological adaptation and networking. Including networking in the background seems a bit like anticipating the conclusions, as the limited attention for network skills in agrifood and forestry masters is one of the main conclusions. Why not mentioning, short, all the seven skill groups. Then also the rest of the abstract becomes more clear.

 The method section in the abstract is rather (too!) limited, important parts of the data analysis are left out (PCA, PLS,…)

 The conclusions section in the abstract gives very limited information. If the remarks on the method of analysis would be left out, there would be space to give more info on the results for the three sectors, and still the important remark on networking skills could be included.

**Introduction**

 Sometimes next to ‘’skills’’, ‘’competencies’’ are mentioned. However, these seem to not be part of the study!?

 The future challenges discussed are not clear; is it about sustainable agri-food and forestry, or are also all kind of other challenges involved? The first part of the introduction section is rather vague.

 Then relating to education programs, it is not clear how the specific skills mentioned relate to the challenges (sustainable production?). For example Newcomb (1993)?. But also how ‘soft skills’, networking, etc. relate to coping with ‘’the challenges’’? For the reader it is very important to know how the skills mentioned, here and further on, in the article help to cope with ‘’the challenges’’. Even a relative short paragraph could help here.

 And also in the next section, what education for sustainability encompasses is rather vague, for example how the type of pedagogy applied relates to sustainability(?) education is not clear. (but this vagueness  in fact holds for the largest part of this paragraph).

**Methods**

 The argumentation to choose Master programs should be improved. Also other agri-food and forestry education levels are relevant and of strategic importance (see e.g. de EU Skills Agenda, 2020).

 Why in the selection of programs >10% STEM students?

 On the selected skill sets, I would have expected a clear relation between figure 1 and table 1. Indeed, it is not transparent how you arrive at table 1. In table 1 soft and  transversal skills seems to be the focus. That these are of high importance is also found in many other studies, so it makes a lot of sense to include these. However, the technical skills in the list focus mostly on computer kills and ''general'' terms like 'technique' etc. Why haven't these technical skill be more specified: think of renewable energy, (value added?) waste management, biodiversity, valorization of by-streams etc etc. (and/or some of the concepts in figure 1).

 It would have made sense to also make a rough divide in the ‘’control’’ group of masters programs, as within this group there will be large differences (e.g. think on the differences between social and natural sciences). Nevertheless, the exercise as performed by the researchers does add to the study!

 The way chosen to validate keywords (with Wikipedia) is creative. However, regarding the statement ‘’ and was based objectively on texts that are generally accepted to be academic and communal descriptions of the three sectors’’, I wouldn’t say that Wikipedia texts are (all) academic and generally accepted.

**Results**

 What is meant by ‘’network analysis’’?

Is the study design appropriate and does the work have academic merit?

Yes

Is the work clearly and accurately presented and does it cite the current literature?

Partly

If applicable, is the statistical analysis and its interpretation appropriate?

I cannot comment. A qualified statistician is required.

Are all the source data underlying the results available to ensure full reproducibility?

Partly

Are the conclusions drawn adequately supported by the results?

Yes

Are sufficient details of methods and analysis provided to allow replication by others?

Partly

Reviewer Expertise:

AgriFood Management and Organisation

I confirm that I have read this submission and believe that I have an appropriate level of expertise to confirm that it is of an acceptable scientific standard, however I have significant reservations, as outlined above.

# Reviewer report: Reviewer response for version 1

**DOI:** 10.21956/openreseurope.18594.r42413

**Published:** 2024-09-25

**Authors:** Gabriella Arcese

**Affiliations:** 1 Università Niccolò Cusano, Roma, Italy

The paper shows a representation of the current state of the master's degree content in Europe, with a focus on agricultural, food and forestry sciences.

 I find the paper very interesting and it fills a gap related to the lack of analysis of the subject in the literature.

 In the abstract, the authors can certainly make improvements regarding the detail of the results.

 In the introduction, I would better specify the results and the structure of the text.

 Results: I would highlight the gap and how it results from this analysis.

 In the discussion part, please emphasize more the interpretation of the output results of these courses.

 any solutions to the problems highlighted

 In general I would try to enrich the scientific literature for comparison where possible.

Is the study design appropriate and does the work have academic merit?

Yes

Is the work clearly and accurately presented and does it cite the current literature?

Yes

If applicable, is the statistical analysis and its interpretation appropriate?

Partly

Are all the source data underlying the results available to ensure full reproducibility?

Yes

Are the conclusions drawn adequately supported by the results?

Partly

Are sufficient details of methods and analysis provided to allow replication by others?

Yes

Reviewer Expertise:

Economy and management

I confirm that I have read this submission and believe that I have an appropriate level of expertise to confirm that it is of an acceptable scientific standard, however I have significant reservations, as outlined above.

# Reviewer report: Reviewer response for version 1

**DOI:** 10.21956/openreseurope.18594.r42418

**Published:** 2024-09-12

**Authors:** Davide Viaggi

**Affiliations:** 1 University of Bologna, Bologna, Italy

The paper provides an interesting investigation about the contents of master courses in Europe, with a focus on agricultural, food and forestry sciences.

 I think the paper is relevant and deserves attention, as the topic of education is not enough studied and investigated in the literature.

 My main major comment is that the rationale of the paper is too much based on a gap in other parts of NEXTFOOD project. This happens in the abstract and throughout the text. I think the reader is not really intrested in this and rather would like to see the background and objectives explained in the context of current literature, policy problems and gaps in research.

 Even when mentioning NEXTFOOD itself, I think the authors should Put more focus on the contents of the skills expressed.

 In the abstract, the authors could improve and enlarge the results with more details; this is the essential part of the exercise. I would modify the introduction based on my previous comment.

 Results: I am not clear what is the meaning of the validation study; in particular reflecting on the differences without expanations does not help in understanding the issue.

 I think the dicussion requires a wider consideration of potential alternative interpretations. As an example, see the discussione on hunger: the current interpretation might work, but there can be others, for example that courses use a wording tending to give a positive feeling (solving problems rather than emphasising the problems themselves). In general this part is rather poor of scientific literature and I think digging a bit more on this would help a better intepretation of the results.

 Also the authors seem not to considerr the different legislation among countries, that is actually a strong driver of course design.

 I could not detect a proper discussion of limitations and biases, that is essential in this cases.

 In the conclusions, I would expect a bit more on the topic and not only consideration about the instrument. To persuade that it is relevant (I am sure it is), I would suggest to spend more time in identifying a few strong insights on the topic.

 Some deliverables from NEXTFOOD are cited. Plese check if the content of deliverables is also in published material, and, in this case, please cite the published version if relevant.

Is the study design appropriate and does the work have academic merit?

Yes

Is the work clearly and accurately presented and does it cite the current literature?

Partly

If applicable, is the statistical analysis and its interpretation appropriate?

Yes

Are all the source data underlying the results available to ensure full reproducibility?

No source data required

Are the conclusions drawn adequately supported by the results?

Partly

Are sufficient details of methods and analysis provided to allow replication by others?

Yes

Reviewer Expertise:

agricultural economics

I confirm that I have read this submission and believe that I have an appropriate level of expertise to confirm that it is of an acceptable scientific standard, however I have significant reservations, as outlined above.
